# Detection of Hepatocellular Carcinoma in a High-Risk Population by a Mass Spectrometry-Based Test

**DOI:** 10.3390/cancers13133109

**Published:** 2021-06-22

**Authors:** Devalingam Mahalingam, Leonidas Chelis, Imran Nizamuddin, Sunyoung S. Lee, Stylianos Kakolyris, Glenn Halff, Ken Washburn, Kristopher Attwood, Ibnshamsah Fahad, Julia Grigorieva, Senait Asmellash, Krista Meyer, Carlos Oliveira, Heinrich Roder, Joanna Roder, Renuka Iyer

**Affiliations:** 1 Robert H. Lurie Comprehensive Cancer Center of Northwestern University, Northwestern University, Chicago, IL 60611, USA; 2 Department of Medicine, Feinberg School of Medicine, Northwestern University, Chicago, IL 60611, USA; imran.nizamuddin@northwestern.edu; 3 Long School of Medicine, University of Texas Health Science Center at San Antonio, San Antonio, TX 78229, USA; halff@uthscsa.edu (G.H.); ken.washburn@osumc.edu (K.W.); 4 Department of Medical Oncology, Democritus University of Thrace, 68100 Alexandroupolis, Greece; leonidas.chelis@kfsh.med.sa (L.C.); skakol@her.forthnet.gr (S.K.); 5 Adult Oncology Department, King Fahad Specialist Hospital Dammam, Dammam 32253, Saudi Arabia; fahad.ibnshamsah@kfsh.med.sa; 6 Department of Medical Oncology, Roswell Park Comprehensive Cancer Center, Buffalo, NY 14203, USA; sslee1@mdanderson.org (S.S.L.); attwood3@buffalo.edu (K.A.); renuka.iyer@roswellpark.org (R.I.); 7 Department of Gastrointestinal (GI) Medical Oncology, The University of Texas MD Anderson Cancer Center, Houston, TX 77030, USA; 8 Department of Surgery, The Ohio State University Wexner Medical Center, Columbus, OH 43210, USA; 9 Biodesix Inc., Boulder, CO 80301, USA; julia.grigorieva@biodesix.com (J.G.); senait.asmellash@biodesix.com (S.A.); krista.meyer@biodesix.com (K.M.); carlos.oliveira@biodesix.com (C.O.); heinrich.roder@biodesix.com (H.R.); joanna.roder@biodesix.com (J.R.)

**Keywords:** cancer screening, cirrhosis, AFP, machine learning, MALDI-TOF, proteomics

## Abstract

**Simple Summary:**

Liver cancer is one of the most common causes of cancer worldwide, but unfortunately, current technology has a limited ability to detect it early in high-risk patients. This study investigates a machine learning algorithm based on protein levels in the blood that can be used to help with diagnosis. The test shows promising results, especially in patients with smaller tumors and compared to current blood detection tests. This research suggests an important role in the future for machine learning algorithm-based blood detection tests.

**Abstract:**

Hepatocellular carcinoma (HCC) is one of the fastest growing causes of cancer-related death. Guidelines recommend obtaining a screening ultrasound with or without alpha-fetoprotein (AFP) every 6 months in at-risk adults. AFP as a screening biomarker is plagued by low sensitivity/specificity, prompting interest in discovering alternatives. Mass spectrometry-based techniques are promising in their ability to identify potential biomarkers. This study aimed to use machine learning utilizing spectral data and AFP to create a model for early detection. Serum samples were collected from three separate cohorts, and data were compiled to make Development, Internal Validation, and Independent Validation sets. AFP levels were measured, and Deep MALDI^®^ analysis was used to generate mass spectra. Spectral data were input into the VeriStrat^®^ classification algorithm. Machine learning techniques then classified each sample as “Cancer” or “No Cancer”. Sensitivity and specificity of the test were >80% to detect HCC. High specificity of the test was independent of cause and severity of underlying disease. When compared to AFP, there was improved cancer detection for all tumor sizes, especially small lesions. Overall, a machine learning algorithm incorporating mass spectral data and AFP values from serum samples offers a novel approach to diagnose HCC. Given the small sample size of the Independent Validation set, a further independent, prospective study is warranted.

## 1. Introduction

Primary liver cancer results in a significant global burden of disease, with studies reporting it as the sixth most common cause of cancer and fourth most common cause of cancer-related death worldwide in 2018. Hepatocellular carcinoma (HCC) makes up 75% to 85% of all primary liver cancers [1]. While reports have suggested a decrease in incidence of HCC in Asia due to vaccination and treatment programs for viral hepatitis, HCC is the fastest growing cause of cancer-related deaths in the United States [2]. Chronic liver disease of any etiology remains the most significant risk factor, with 80% to 90% of new HCC cases occurring in this population [3]. Surveillance programs have been developed for earlier detection and mortality reduction. Current AASLD guidelines recommend surveillance in adults with cirrhosis and high-risk patients without cirrhosis using ultrasound with or without alpha-fetoprotein (AFP) assessment at six-month intervals [4]. Unfortunately, screening ultrasound may be of limited use among select populations secondary to body habitus, obesity, early HCC disease, and operator experience [5]. In such cases, biomarkers may supplement ultrasound in the detection of early disease. However, the sensitivity and specificity of AFP is barely satisfactory, necessitating the discovery of circulating biomarkers with a higher diagnostic value [6]. In fact, neither European nor American guidelines include quantification of serum AFP for HCC diagnosis, despite estimated improvement of 6% to 8% in detection rate. Reasons for its suboptimal performance include lack of sensitivity for detecting hepatocellular carcinoma in early stages and large numbers of false-positive results [7].

Several candidate biomarkers are being studied for HCC diagnosis, with des-gamma-carboxy prothrombin (DCP), lens cullinaris agglutin-reactive AFP (AFP-L3), osteopontin, and midkine, amongst others, the most advanced in development. Nevertheless, significant challenges exist, largely stemming from HCC molecular heterogeneity [8]. Furthermore, many of these biomarkers continue to be plagued with low sensitivity, especially when used without AFP [9]. Certain biomarkers, such as DCP and AFP-L3, are markers of advanced tumoral stage, thus preventing their use for early cancer detection [10,11]. Recognizing that HCC tumor biology is highly heterogeneous, composites of biomarkers and clinical factors associated with risk of HCC have been investigated for early detection of HCC. One such panel, the GALAD score, uses objective measures of gender, age, AFP, AFP-L3, and DCP [12]. The sensitivity/specificity of GALAD at a fixed cutoff of −0.63 has ranged from 92%/90%, 71%/96%, and 88%/89% in cohorts from the UK, Japan, and Germany [13] to 79%/79% in a cohort from the USA [14].

Recently developed mass spectrometry-based techniques, such as proteomics, lipidomics, and metabolomics, represent promising tools for the discovery and identification of proteins, peptides, lipids, and metabolites associated with various diseases [15]. Among various mass spectrometric techniques, matrix-assisted laser desorption/ionization time-of-flight (MALDI-TOF) mass spectrometry is a high-throughput technology capable of generating a molecular fingerprint. Thus, it has provided a powerful tool for discovery of biomarkers in different kinds of cancers, including HCC [16,17,18]. However, traditionally matrix-assisted laser desorption/ionization (MALDI)-based studies have been hampered by lack of sensitivity. A new approach, the Deep MALDI^®^ method, which averages over many more laser shots than conventional methods, allows for a deeper probing of the serum proteome [19]. Machine learning (ML) techniques have been applied to combine MALDI mass spectral (MS) data with clinical data to generate molecular diagnostic tests predictive of outcomes for cancer therapy [20,21].

Herein, we propose using this technology for test development and blinded validation on three independent sample sets from healthy volunteers, patients with known cirrhosis without HCC, and patients with HCC. The main goal of the study is to identify a signature of early HCC among patients with cirrhosis or high-risk patients with chronic liver disease. We focus on the assessment of test performance in the patients with the smallest lesions, where early detection and intervention is most important.

## 2. Materials and Methods

### 2.1. Patient Cohorts

Two patient cohorts were used for test development and initial validation: a cohort of 100 pre-transplant patients (48 HCC and 52 cirrhosis) from University of Texas Health Sciences Center San Antonio (UTHSCSA) and a cohort of 193 patients (110 HCC and 83 cirrhosis) from Democritus University of Thrace, Greece (Greek). A third cohort of 156 patients (97 HCC and 59 healthy volunteers) from Roswell Park Comprehensive Cancer Center (Roswell) was used for blinded, independent validation of the test. Serum samples had been collected from patients in the UTHSCSA cohort at time of liver transplant. Blood collection protocols were approved by the respective institutional review committees, and patient consent was obtained. The study conformed to ethical guidelines of the 1975 Declaration of Helsinki.

Of the UTHSCSA cohort containing 100 patients, 48 patients had HCC and 52 patients had liver disease without HCC. Patients undergoing liver transplant for HCC generally had much better liver function than those with other liver diseases. The predominant liver disease etiologies across all 100 patients were alcohol-related cirrhosis and hepatitis C. The Greek cohort consisted of 110 patients with HCC and 83 patients with liver disease without HCC. Within this cohort, 68% of patients had hepatitis B. The Roswell cohort consisted of 97 patients with HCC and 59 healthy volunteers without HCC, totaling 156 patients.

**Figure 1:**
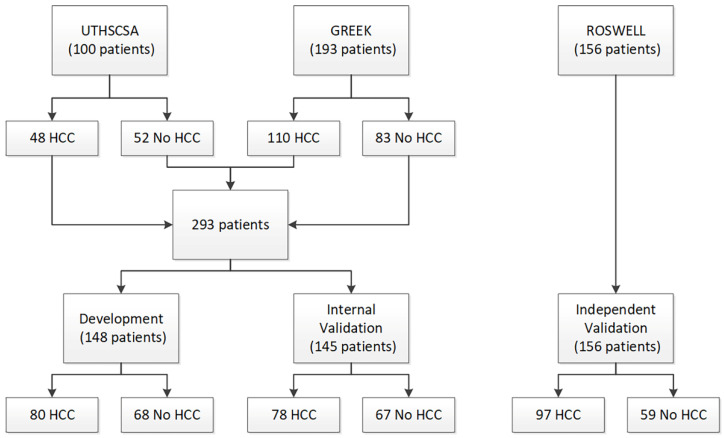
Consort diagram.

As there were differences in liver function and liver disease etiology between the two cohorts used for test development, the UTHSCSA and Greek cohorts were combined, split, and stratified by presence/absence of HCC to create a Development set and an Internal Validation set (Figure 1). All test development work was carried out using data from only the Development set. Patient characteristics for all three cohorts and the Development set and the Internal Validation set are provided in Table 1.

### 2.2. Methods

#### 2.2.1. Sample Collection and Storage

Serum samples were stored at −80 °C and were shipped frozen in batches to the Biodesix laboratory (Biodesix, Boulder, CO, USA) for MS generation and AFP measurement.

#### 2.2.2. Mass Spectral Acquisition

In total, 3 μL aliquots of each experimental sample were sufficient for generation of mass spectra. To simulate sample collection procedures practical for clinical use with sample shipment at ambient temperature, serum samples were spotted onto cellulose serum cards (Therapak, Claremont, CA, USA), allowed to dry, and then re-eluted. Spectra were obtained using a MALDI-TOF mass spectrometer (Ultraflextreme, Bruker, Billerica, MA, USA). The Deep MALDI^®^ method was used, providing data over a greater dynamic range than standard MALDI approaches [19]. Eight hundred shot spectra were collected from 63 pre-defined positions per MALDI spot (63 × 800 × 3 spots per sample), for a total of 151,200 laser shots per sample. Spectra were collected from the UTHSCSA cohort in November 2013, the cohort of 16 patients with no liver disease in July 2014, the Greek cohort in March 2015, and the Roswell cohort in February 2018.

#### 2.2.3. Mass Spectral Processing

All spectra were aligned (Table S1) and spectra failing quality control metrics were discarded. At random, 140 spectra for each sample were selected and averaged to create one average spectrum (from 112,000 laser shots) per sample. Average spectra then underwent processing to make them comparable between samples (Figure S1). This involved background subtraction, normalization (Table S2, Table S5), batch correction using spectral data from reference samples, and alignment (Table S3). Full details of sample preparation and spectral processing methods are provided in Supplementary Text S1.

Three hundred mass spectral features were defined (Table S4). Each MS feature is defined as a mass/charge region and the value of a MS feature is the integrated intensity of the processed, average spectrum within this mass/charge region. MS feature values were calculated for each processed averaged spectrum for each sample.

#### 2.2.4. AFP Measurement

Serum AFP levels were measured for each sample using the DAFP00 ELISA kit (R&D Systems, Minneapolis, MN, USA) following manufacturer instructions as described in Supplementary Text S1 by ELISA Tech (Aurora, CO, USA).

#### 2.2.5. Application of an Existing MS-Based Serum Proteomic Test

The classification algorithm from a pre-existing serum proteomic test (the VeriStrat^®^ test, Biodesix, CO, USA) was applied to the generated mass spectra [16]. This test produces a binary classification of Good or Poor and has been demonstrated to have prognostic and predictive utility in advanced non-small cell lung cancer [22]. It has been observed that Poor classifications are rarely observed in patients without cancer [23].

#### 2.2.6. Development of the HCC Detection Test

1. Machine Learning Approach

Test development was carried out using machine learning with a dropout regularized combination (the Diagnostic Cortex^®^ system, Biodesix., Boulder, CO, USA) approach [24]. This method was designed to allow reliable estimates of test performance from relatively small development sets in the setting where there are more measured attributes than samples. Briefly, the Development set was divided into a training set and test set. The 300 MS features and AFP were used as attributes to classify the samples into “Cancer” or “No Cancer” groups. Many simple, k-nearest neighbor, atomic classifiers were constructed with the training set using subsets of the attributes. Atomic classifiers not showing any ability to correctly classify the training set samples were discarded during a filtering step. The remaining atomic classifiers were combined using dropout regularized logistic regression to yield one master classifier. This was repeated for many splits of the Development set into training and test sets, and an ensemble average was created to generate a final score for each sample. As each sample was held in the test set for multiple training/test split stratifications, reliable classification estimates could be obtained for all samples in the Development set by ensemble averaging only test set data (out-of-bag estimation). Application of a threshold to the resulting score yielded a binary classification of “Cancer” or “No Cancer” for each sample. The family of tests produced from varying the threshold value was assessed using receiver operating characteristic (ROC) methods. A final test was produced by choice of a particular threshold best suiting clinical need in terms of its associated sensitivity and specificity.

2. Test Development

**Figure 2:**
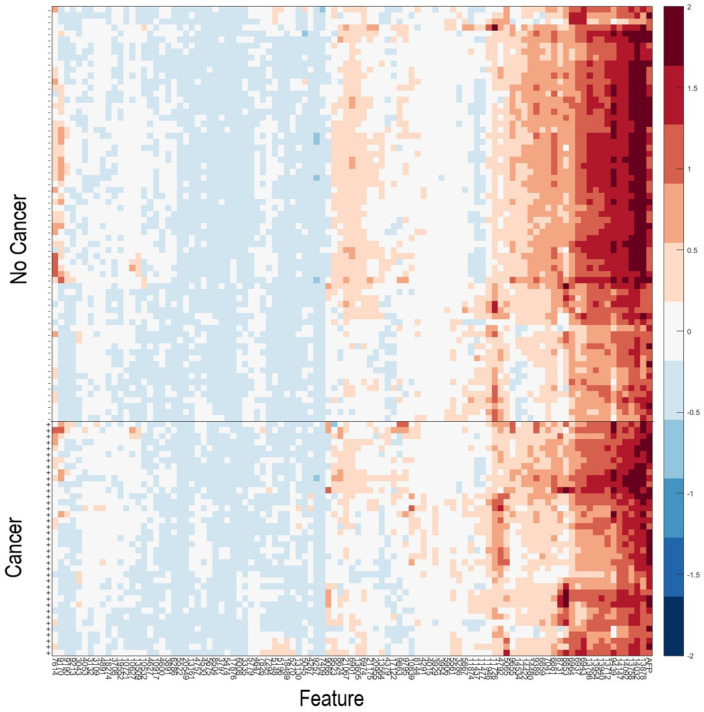
Heatmap of the natural logarithm of serum AFP and the MS features used for classification for the 108 samples used in classifier development. + indicates samples from patients with HCC; -indicates samples from patients without HCC. Features and samples are hierarchically clustered.

As it has been observed that patients with serum samples classified as Poor by the VeriStrat^®^ classification algorithm or with very high AFP are very likely to have cancer, patients meeting these criteria (*n* = 40) were assigned a “Cancer” classification. Data for the remaining samples (*n* = 108) in the Development set were then used within the machine learning platform for training of a classifier able to identify patients with or without HCC, based on their serum AFP and values of the 100 mass spectral features showing the greatest potential for classification (Table S6). Figure 2 shows a heatmap of the 100 MS features used within the classification algorithm for the 108 samples used in classifier development, grouped according to “Cancer” vs. “No Cancer”. A list of the feature definitions of the 100 MS features and assessment of the univariate associations of the features with presence or absence of HCC is contained in the Supplementary Text S1. It is noteworthy that no single feature provided outstanding classification alone. We observed that some pairs of features, which individually had relatively poor classification power, provided much better classification as an interaction (i.e., product of the two), indicating the multivariate nature of the test.

Imbalances between the liver function of patients with HCC and without HCC were observed in our cohorts, as evidenced by MELD and Child–Pugh scores. This was particularly apparent in the UTHSCSA cohort. Samples were collected at the time of transplant or resection. Hence, patients without HCC eligible for a liver transplant had very advanced liver disease with associated poor liver function, while patients undergoing transplant or resection for early stage HCC had better liver function, typical of the population at risk for HCC (Table 1). Liver function is easily assessable from measurements of the serum proteome, and serum mass spectra for patients with poor liver function display many differences from those for patients with better liver function. Hence, our data were partially confounded. The dropout regularized combination approach of test development is well-suited to mitigate such confounding effects [24]. In addition to requiring that atomic classifiers had a minimal level of performance classifying the training set, we required that they also were able to classify spectra from serum of healthy patients to the “No Cancer” group. More details on machine learning classifier development are provided in Supplementary Text S1.

All test parameters, including the threshold for the binary result of “Cancer” or “No Cancer” were set using only samples from the Development set and locked prior to all validation.

#### 2.2.7. Application of the HCC Detection Test to Validation Samples

**Figure 3:**
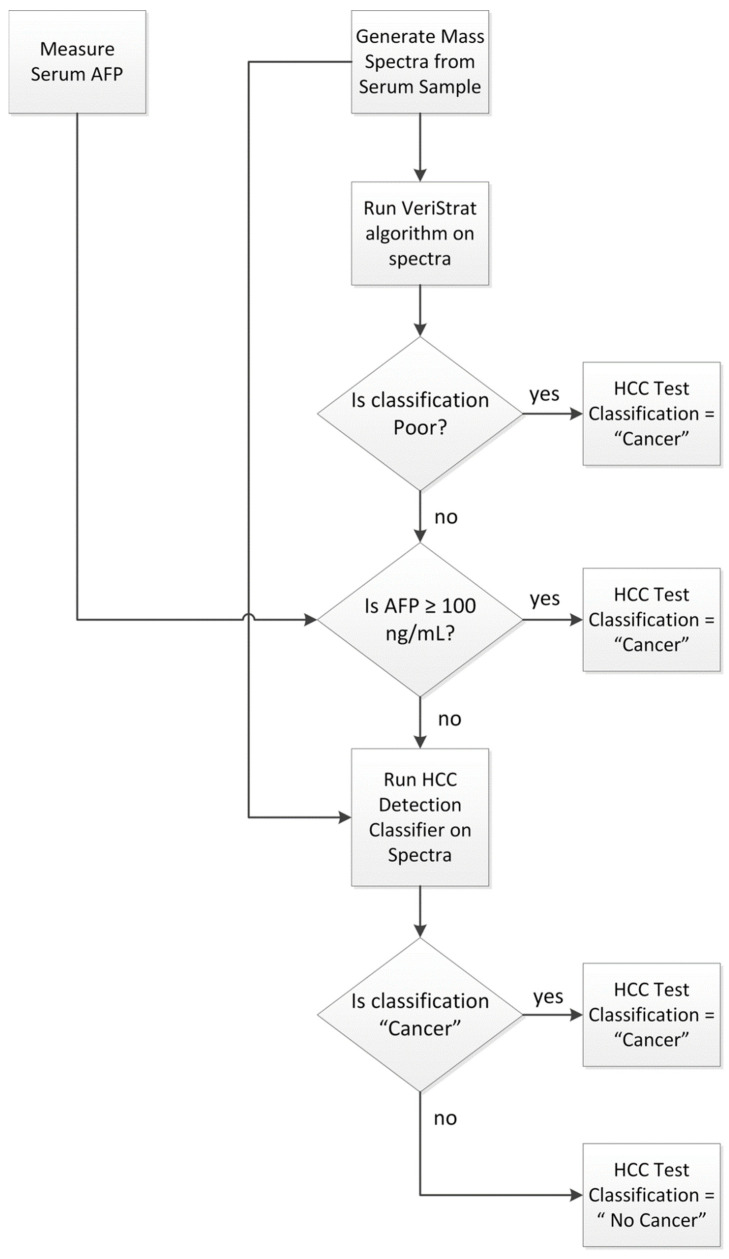
Classification algorithm for the HCC Detection Test.

The HCC detection test was applied to any sample not used in its development following the schema of Figure 3.

First, mass spectra were acquired from the serum sample, and serum AFP was assessed following the protocols outlined above and in Supplementary Text S1. The VeriStrat classification algorithm was then applied to the generated mass spectra and samples yielding a Poor classification were assigned a “Cancer” classification. Samples with serum AFP determined as equal to or exceeding 100 ng/mL were also assigned a “Cancer” classification. Samples not yielding a VeriStrat Poor classification and with AFP < 100 ng/mL were then classified as “Cancer” or “No Cancer” by the machine learning classifier, based on their MS feature values and serum AFP measurement. Quality control metrics were applied to the MS data, so that only samples generating mass spectra of sufficient quality and not exhibiting evidence of sample contamination or degradation received a valid test classification.

#### 2.2.8. Independent Validation

Independent validation was performed using the fully locked test. Mass spectra were generated from samples in the Roswell validation set more than 2 years after collection of spectra used in test development and were classified blinded to all clinical data.

#### 2.2.9. Statistical Methods

Analyses were performed using SAS 9.3 (SAS, Cary, NC, USA) and PRISM (GraphPad, La Jolla, CA, USA). The area under the curve obtained from the test of Figure 3 was compared with that obtained from AFP alone using the method of DeLong. Test performance was assessed using sensitivity, specificity, and accuracy of detection of HCC within patient subgroups.

## 3. Results

Analysis of the spectra from the Development set accurately classified 66 of 80 (83% sensitivity) HCC specimens and 57 of 68 (84% specificity) non-HCC specimens. Of the Internal Validation set, 63 of 78 (81% sensitivity) HCC specimens and 53 of 67 (79% specificity) non-HCC specimens were accurately classified. Finally, of the Independent Validation set, 85 of 97 (88% sensitivity) HCC specimens and 59 of 59 (100% specificity) non-HCC specimens were accurately classified.

**Figure 4:**
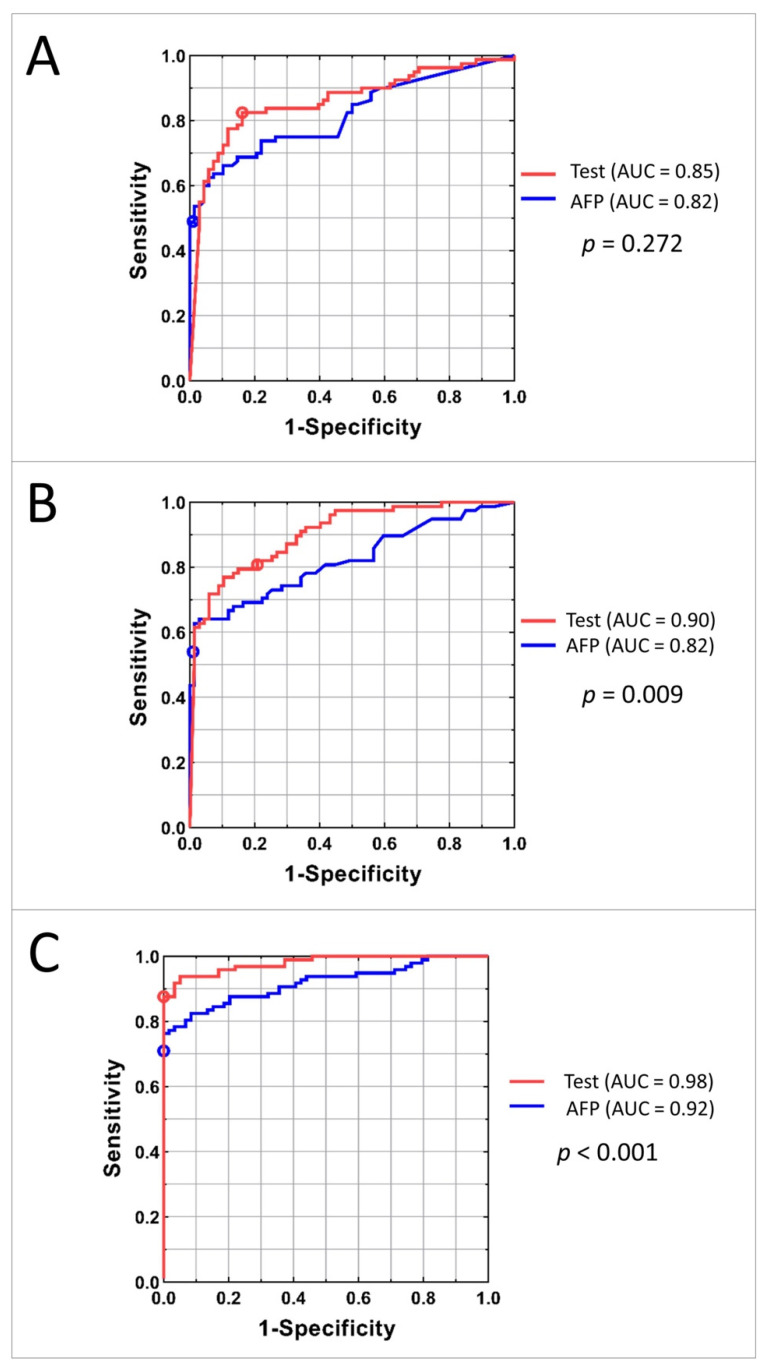
AUC curves for Development (**A**), Internal Validation (**B**), and Independent Validation (**C**) sets. Red line represents the family of tests obtained by adjusting the cutoff of the classifier; the red circle shows the results for the test with specified cutoff. Blue line corresponds to the classification using AFP concentration; blue circle shows the results for the AFP cut-off 20 ng/mL.

To compare the classification power of the family of tests obtained by varying the threshold applied to the HCC test classifier with that of serum AFP level, ROC plots were constructed. The ROC plots for the Development, Internal Validation, and Independent Validation sets for AFP alone and the tests using mass spectrometry data and AFP are shown in Figure 4. P values for comparison of the area under the curves (AUCs) between the test and the AFP classification are shown on the right.

The similarity of AUCs between the Development and Internal Validation sets indicates excellent generalization of classification performance. Increased performance in the Independent Validation set is likely due to the differences in population. The test showed significantly better performance than univariate AFP level in both Internal and Independent Validation sets. In the Independent Validation set, at sensitivity of 88%, the test specificity exceeded that of univariate AFP by 20%. At perfect specificity, the test sensitivity exceeded that of univariate AFP by 13%.

Diagnostic performance of the commonly used cut-off for AFP of 20 ng/mL typically produces sensitivities in the range of 41–65% and specificities of 80–90%, depending on patient population [25,26]. In our study, detection of HCC by the 20 ng/mL cut-off (marked on the ROC curves in Figure 4) resulted in sensitivities of 49%, 54%, and 71% in the Development, Internal Validation, and Independent Validation sets, respectively, which is markedly inferior to the results of the test. Specificity of using AFP cut-off as a biomarker was high: 99%, 99%, and 100% in the respective cohorts.

Accuracy of test classification for patients with and without cancer in each of the cohorts overall and in clinical subgroups defined by liver function, origin of the disease, and lesion size is shown in Table 2.

The test demonstrated high specificity, independent of cause and severity of underlying liver disease in the Internal Validation set. The test also showed excellent specificity in the Independent Validation set, indicating that the utility is not restricted to patients with impaired liver function or advanced liver disease. The results for HCC patients depending on cancer stage and lesion size confirm high sensitivity of the test for early stages and small tumors (Table 3).

**Figure 5:**
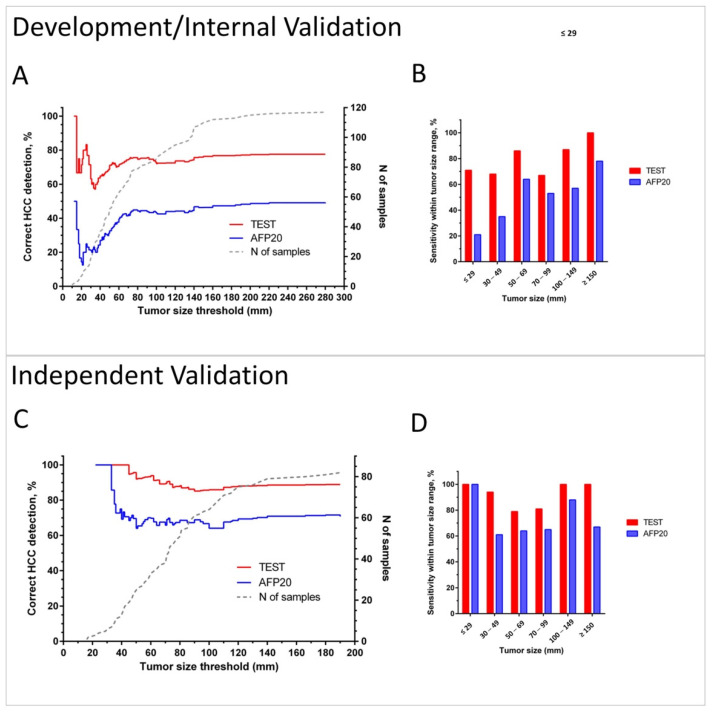
Sensitivity of HCC detection by the test and by AFP using cut-off 20 ng/mL (AFP20) in cancer patients. Plots A and C show in red (Test) and blue (AFP20) lines % of correct identifications of HCC for patients with tumors up to the threshold tumor size in the combined Development/Internal Validation set (**A**) and in the Independent Validation set (**C**). Dotted grey line (corresponding to the right Y-axis) shows the number of patients with tumors up to the threshold size. Bar charts B and D show sensitivity within the selected tumor size ranges in the Development/Internal Validation set (**B**) and in the Independent Validation set (**D**).

Figure 5 illustrates the sensitivity of the test in the combined Development/Internal Validation sets and in the Independent Validation set depending on tumor size in comparison with the detection by applying the 20 ng/mL AFP concentration cut-off.

Cumulative plots (Figure 5A,C) and bar charts of sensitivity for groups within selected tumor size ranges (Figure 5B,D) show that the test has improved cancer detection compared to AFP for all tumor sizes and independently of the chosen tumor size threshold. The advantage of the test is especially pronounced in diagnosis of small lesions. It detected 69% of HCC cases in BCLC A overall, 71% lesions <3 cm in combined Development and Internal Validation sets, and 100% of tumors <3 cm in the Independent Validation set. The test also correctly diagnosed 75% and 76% of grade I and II tumors, as well as 75% and 86% of HCC in stage I and II patients in the Independent Validation set (see Table S7).

## 4. Discussion

MALDI-TOF has been a promising tool for the identification of serum biomarkers in many cancers [27]. This technology has been applied to identify proteins, peptides, and metabolites related to gastrointestinal, lung, prostate, renal, breast, ovarian and hematological cancers [27,28,29]. Furthermore, it has been combined with ML to create algorithms for both diagnosis and patient stratification for cancer therapy [20,21,30]. The utility of ML algorithms for detection of HCC or staging of chronic liver disease has also been explored [31,32,33], although independent validation of the results is generally not yet available. Many such studies have relied on spectra generated from liver specimens, which are difficult to acquire [32,33,34]. Thus, HCC detection tests utilizing serum markers, as in this current study, have great potential for accessible use in the clinical setting.

Our current work set out to identify a robust signature of early HCC among high-risk patients with chronic liver disease or cirrhosis and assess its performance in blinded validation using serum samples from healthy volunteers, patients with known cirrhosis, and patients with HCC. Our model incorporates both AFP measurements and MS-based proteomics in a ML algorithm. Overall, the HCC detection test had greater sensitivity and specificity compared to AFP alone and showed significantly better performance than AFP alone in both Internal and Independent Validation sets. Even with differences in patient demographics, test performance was consistent across Development, Internal Validation, and Independent Validation sets. This is despite the collection of the retrospective sample sets under independent protocols, at different institutions and geographic regions, and with the samples run over a period of several years. These observations point toward the generalizability of the ML-based test and the stability and reproducibility of the MS data obtained. The test was able to detect HCC with a sensitivity of 81% or greater at specificity of 79% or above in all three cohorts. In the Independent Validation set, at a sensitivity of 88%, the test specificity exceeded that of univariate AFP by 20%. It is noteworthy that test specificity was high even though the cancer-free subjects in each cohort represented clearly different populations in all three cohorts (liver transplant candidates in the UTHSCSA cohort, high prevalence of hepatitis B in the Greek cohort, and subjects with healthy livers in the Roswell cohort). Moreover, test sensitivity was high across liver disease etiology, including hepatitis B and hepatitis C.

According to a systematic review, AFP with a cut-off value of 20 ng/mL (AFP20) had a sensitivity and specificity of 49% to 71% and 49% to 86%, respectively [6]. However, this analysis included mainly Asian studies with tumor size < 5 cm. In comparison, AFP20 sensitivity in this current study was 49% to 71%, in line with two recently analyzed US cohorts [14], while we found higher specificity in our cohorts of 99% to 100%. At a cutoff of −0.63, GALAD score has demonstrated sensitivity/specificity of 92%/90% in a UK cohort [13] and 79%/79% in a US cohort [14], the EDRN multicenter cohort of 545 subjects. Differences in cohorts make comparison of test performance across studies extremely difficult. Unfortunately, AFP-L3 and DCP levels were not available for our patients, precluding a direct comparison of the performance of our test with GALAD scores. Future studies designed to assess these biomarkers in addition to our HCC detection test would be necessary to reliably establish relative performance and whether MS data can provide information that could supplement these scores.

Early diagnosis of HCC, when resection or intervention may still be possible, is crucial, given that the overall prognosis is dismal, with a 5-year survival rate of less than 10%. By the time of diagnosis, only 20% to 30% of patients are eligible for curative therapy, e.g., with transplantation, surgical resection, or local ablative processes [35]. In our study, the HCC detection test was able to recognize early stages of HCC. Notably, it detected 69% of HCC cases in BCLC A overall, 71% lesions <3 cm in combined Development and Internal Validation sets, and 100% of tumors <3 cm in the Independent Validation set. While AFP20 had similar performance for the smallest tumors in our Independent Validation set, its performance was markedly worse in the Development/Internal Validation, and our test detection rates were much higher than those reported for traditional univariate biomarkers, such as AFP, AFP-L3, and DCP, when used for early detection [6].

Moreover, our test was performed both in cirrhotic and non-cirrhotic patients, demonstrating excellent specificity both for Internal and Independent Validation sets regardless of the underlying liver impairment status. While diagnosis of HCC for cirrhotic patients can be established by imaging criteria using LI-RADS classification [36], diagnosis is more difficult in non-cirrhotic patients given that the LI-RADS score cannot be applied and tissue biopsy is mandatory to establish the diagnosis. Therefore, in the future, after prospective validation, our test may help in establishing HCC diagnosis for non-cirrhotic patients without the need for an invasive tissue biopsy procedure.

While the results of our study are promising, there are several weaknesses. First, the study was retrospective, which introduces possibilities for confounding, and some demographic data from all cohorts were incomplete. Liver function was dramatically different between the patients with HCC in the UTHSCSA and Greek cohorts, which led to these cohorts being combined and then split in two to generate a suitable Development set. The Independent Validation set included only 156 patients, and the subjects without HCC were not representative of patients at high risk of developing HCC. A larger scale validation in patients at high risk of developing HCC would be helpful to ensure generalizability of the results in the most relevant population. Second, in our study, AFP performed surprisingly well in detection of hepatocellular carcinoma, with AFP20 demonstrating very high specificity. Even though AFP had AUCs ranging from 0.82 to 0.92 (Figure 4), we still observed significant differences in AUCs between AFP alone and the HCC detection test. While the high specificity of AFP in the Independent Validation set may be due to it consisting of healthy volunteers, the reason for the good performance of AFP in the other cohorts is not readily apparent. Ongoing validation with larger sample sizes and a range of control populations is needed. Lastly, as in most other studies, the number of patients with early stage cancer in our cohorts was small, making it hard to accurately assess test sensitivity in this population, where improved detection could be most beneficial.

Ultimately, the goal of this study was to use high-throughput MS-based techniques to discover a serum signature for early HCC detection. The high-throughput nature of MALDI mass spectrometry and the use of cards to allow ambient temperature shipment of dried serum make the test practical in a clinical setting. Indeed, the VeriStrat MALDI-based MS test, as used in clinical practice for assessment of patients with NSCLC, uses overnight ambient shipping of dried blood-based samples to a centralized laboratory. However, given that the signature is composed of unidentified features, clinicians would need to become comfortable with relying on a result determined by the relative expression levels of certain proteins without recognizing any obvious mechanistic basis. Nevertheless, this limitation also applies to several biomarker profiles, including the GALAD score, as well as other MS-based approaches. Future studies may further explore combining other protein biomarkers and patient characteristics with AFP and Deep MALDI mass spectral data using ML methods. New approaches to explainability of machine learning algorithms, protein identification of the most important MS features used for classification, and translational studies comparing patients correctly or incorrectly classified by the test may be useful to increase physician trust in the test.

Future studies may further explore combining other protein biomarkers and patient characteristics, such as age, gender, and liver disease etiology, with AFP and Deep MALDI mass spectral data using modern machine learning methods. Incorporating MS data into existing, validated serological models (i.e., GALAD scores) may further contribute to accurate diagnosis. A prospective trial in high-risk populations, including increased numbers of patients with early stage disease, is necessary for further validation, comparison with the GALAD score and other novel HCC detection tests, as well as determination of clinical utility.

## 5. Conclusions

In summary, the results for our HCC detection test are positive, with impressive sensitivity and specificity, especially on the Independent Validation set with blinded validation. The test was able to identify small tumors in early stages, comparing favorably to currently used biomarker panels. Lastly, the test was conducted on human serum, greatly improving accessibility compared to HCC detection tests requiring liver biopsy samples. Nevertheless, work remains to be carried out prior to adoption of the test in clinical practice. A prospective trial in high-risk populations is necessary for further validation, comparison with other validated scores, and assessment of generalizability and clinical utility.

## Figures and Tables

**Table 1:** Patient characteristics by cohort and set.

Patient Characteristic		Cohort						Set			
		UTHSCSA		Greek		Roswell		Development		Internal Validation	
		HCC (*n* = 48)	No HCC (*n* = 52)	HCC (*n* = 110)	No HCC (*n* = 83)	HCC (*n* = 97)	No HCC (*n* = 59)	HCC (*n* = 80)	No HCC (*n* = 68)	HCC (*n* = 78)	No HCC (*n* = 67)
Age	median	59.5	56	69	54	63	62	67	54.5	66	57
	range	50–85	40–67	44–82	28–80	38–89	38–87	44–82	30–74	47–85	28–80
Gender	male, *n* (%)	32 (67)	25 (48)	92 (84)	60 (72)	84 (87)	40 (68)	63 (79)	42 (62)	61 (78)	43 (64)
	female, *n* (%)	16 (33)	27 (52)	18 (16)	23 (28)	13 (13)	19 (32)	17 (21)	26 (38)	17 (22)	24 (36)
MELD	median	14	25	10	NA	11	NA	11	25	11	25
	range	7–37	13–47	6–26	NA	6–38	NA	6–34	16–42	7–37	13–47
	NA, *n* (%)	13 (27)	0 (0)	2 (2)	83 (100)	14 (14)	59 (100)	7 (9)	42 (62)	8 (10)	41 (61)
Child-Pugh	A, *n* (%)	30 (63)	6 (12)	72 (65)	74 (89)	53 (55)	NA	35 (44)	38 (56)	37 (47)	36 (54)
	B, *n* (%)	16 (33)	38 (73)	27 (25)	7 (8)	24 (25)	NA	16 (20)	3 (4)	11 (14)	4 (6)
	C, *n* (%)	2 (4)	8 (15)	11 (10)	2 (2)	6 (6)	NA	5 (6)	1 (1)	6 (8)	1 (1)
	NA, *n* (%)	0 (0)	0 (0)	0 (0)	0 (0)	14 (14)	NA	24 (30)	26 (38)	24 (31)	26 (39)
BCLC status	A, *n* (%)	48 (100)	NA	3 (3)	NA	29 (30)	NA	26 (33)	NA	25 (32)	NA
	B, *n* (%)	0 (0)	NA	15 (14)	NA	12 (12)	NA	9 (11)	NA	6 (8)	NA
	C, *n* (%)	0 (0)	NA	73 (66)	NA	41 (42)	NA	35 (44)	NA	38 (49)	NA
	D, *n* (%)	0 (0)	NA	19 (17)	NA	15 (15)	NA	10 (13)	NA	9 (12)	NA
Liver disease	HBV, *n* (%)	4 (8)	1 (2)	72 * (65)	59 (71)	3 (3)	3 (5)	41 (51)	29	35 * (45)	31
origin	HCV, *n* (%)	28 (58)	21 (40)	10 * (9)	7 (8)	26 (27)	13 (22)	18 (23)	15	20 * (26)	13
	Other/NA, *n* (%)	17 (35)	30 (58)	29 (26)	17 (20)	68 (70)	43 (73)	21 (26)	24	24 (35)	23
Serum AFP	median	4.7	1.6	37.0	2.0	4.0	2.6	16.8	1.8	25.0	2.1
(ng/mL)	minimum	<0.8	<0.8	1.1	0.8	<1.5	<1.5	<1.5	<1.5	<0.8	<0.8
	maximum	≥10,000	15.0	≥10,000	115	≥10,000	11.5	≥10,000	20.0	≥10,000	115
Lesion size	<3, *n* (%)	13 (27)	NA	1 (1)	NA	5 (5)	NA	8 (10)	NA	6 (8)	NA
(cm)	≥3 and <5, *n* (%)	21 (44)	NA	13 (12)	NA	18 (19)	NA	16 (20)	NA	18 (23)	NA
	≥5 and <7, *n* (%)	4 (8)	NA	18 (16)	NA	14 (14)	NA	12 (15)	NA	10 (13)	NA
	≥7 and <10, *n* (%)	3 (6)	NA	12 (11)	NA	26 (27)	NA	7 (9)	NA	8 (10)	NA
	≥10 and <15, *n* (%)	2 (4)	NA	21 (19)	NA	16 (16)	NA	12 (15)	NA	11 (14)	NA
	≥15, *n* (%)	2 (4)	NA	7 (6)	NA	3 (3)	NA	4 (5)	NA	5 (6)	NA
	NA, *n* (%)	3 (6)	NA	38 (34)	NA	15 (15)	NA	21 (26)	NA	20 (26)	NA

* One patient had both hepatitis B and hepatitis C; Abbreviations: not available (NA), hepatocellular carcinoma (HCC), Model for End-Stage Liver Disease (MELD), Barcelona-Clinic Liver Cancer (BCLC), alpha-fetoprotein (AFP), hepatitis B virus (HBV), hepatitis C virus (HCV).

**Table 2:** Accuracy of the test overall and by clinical subgroups depending on liver function and origin of the disease.

Cohort		Development (*n* = 148)		Internal Validation (*n* = 145)		Independent Validation (*n* = 156)	
		HCC (*n* = 80)	No HCC (*n* = 68)	HCC (*n* = 78)	No HCC (*n* = 67)	HCC (*n* = 97)	No HCC (*n* = 59)
**Overall, *n* (%)**		66/80 (83)	57/68 (84)	63/78 (81)	53/67 (79)	85/97 (88)	59/59 (100)
Child–Pugh	A, *n* (%)	28/35 (80)	35/38 (92)	30/37 (81)	34/36 (94)	unknown	N/A
	B, *n* (%)	16/16 (100)	3/3 (100)	11/11 (100)	2/4 (50)	unknown	N/A
	C, *n* (%)	5/5 (100)	1/1 (100)	6/6 (100)	1/1 (100)	unknown	N/A
	NA, *n* (%)	17/24 (71)	18/26 (69)	16/24 (67)	16/26 (62)	unknown	N/A
Liver Disease Origin *	HBV, *n* (%)	36/41 (88)	28/29 (97)	31/35 (89)	29/31 (94)	3/3 (100)	N/A
	HCV, *n* (%)	14/18 (78)	10/15 (67)	18/20 (90)	8/13 (62)	26/26 (100)	N/A
	Other/NA, *n* (%)	20/26 (77)	21/26 (81)	18/27 (67)	18/26 (69)	56/68 (82)	N/A

* One patient had both hepatitis B and hepatitis C; Abbreviations: not available (N/A).

**Table 3:** Sensitivity of the test in HCC subgroups depending on tumor size and BCLC status.

Category		Cohort		
		Development (*n* = 80)	Internal Validation (*n* = 78)	Independent Validation (*n* = 97)
BCLC status	A, *n* (%)	19/26 (73)	16/25 (64)	unknown
	B, *n* (%)	4/9 (44)	3/6 (50)	unknown
	C, *n* (%)	33/35 (94)	35/38 (92)	unknown
	D, *n* (%)	10/10 (100)	9/9 (100)	unknown
Lesion size (cm)	<3, *n* (%)	6/8 (75)	4/6 (67)	5/5 (100)
	≥3 and <5, *n* (%)	12/16 (75)	11/18 (61)	17/18 (94)
	≥5 and <7, *n* (%)	10/12 (83)	9/10 (90)	11/14 (79)
	≥7 and <10, *n* (%)	5/7 (71)	5/8 (63)	21/26 (81)
	≥10 and <15, *n* (%)	10/12 (83)	10/11 (91)	16/16 (100)
	≥15, *n* (%)	4/4 (100)	5/5 (100)	3/3 (100)
	NA, *n* (%)	19/21 (90)	19/20 (95)	12/15 (80)

## Data Availability

The data that support the findings of this study are available from the corresponding author, D.M., upon reasonable request.

## Supplementary Materials

The following are available online at https://www.mdpi.com/article/10.3390/cancers13133109/s1, Text S1, Table S1: Points in *m*/*z* used to align the raster spectra, Table S2: Normalization windows used in pre-processing the spectra, lower and upper *m*/*z* boundaries, Table S3: Alignment points used to align the Deep MALDI average spectra, Table S4: Definitions of mass spectral features, Table S5: *m*/*z* windows used in the final feature value normalization, Table S6: Features used in classification (rounded to nearest Dalton for MS features) with measures of their univariate association with presence/absence of HCC in the classifier development set of patients (n = 108, no VeriStrat Poor classification and AFP < 100 ng/mL), Table S7: Accuracy of detection of HCC in Independent Validation (n = 97) by grade and stage, Figure S1: Spectra generated from serum from a patient with HCC (in red) and a patient without HCC (in blue).
