# Supplementary material for: Detection of Hepatocellular Carcinoma in a High-Risk Population by a Mass Spectrometry-Based Test
Source: Cancers (Basel). 2021 Jun 22;13(13):3109. doi: 10.3390/cancers13133109 (PMC8268628; doi:10.3390/cancers13133109)
Supplement: Supplementary file 1 [file cancers-13-03109-s001.zip › cancers-1254849-supplementary.pdf]

Supplementary Materials: Detection of Hepatocellular Carcinoma in a High-Risk Population by a Mass Spectrometry-Based Test

Devalingam Mahalingam, Leonidas Chelis, Imran Nizamuddin, Sunyoung S. Lee, Stylianos Kakolyris, Glenn Halff, Ken Washburn, Kristopher Attwood, Ibnshamsah Fahad, Julia Grigorieva, Senait Asmellash, Krista Meyer, Carlos Oliveira, Heinrich Roder, Joanna Roder and Renuka Iyer

Text S1

1. Acquisition of Mass Spectra from Serum Samples

Processing of Serum Samples

Samples were thawed and 3 μL aliquots of each experimental sample and quality control reference serum, a pooled sample obtained from serum from five healthy patients purchased from ProMedDx (Norton, MA, USA) were spotted onto VeriStrat® serum cards (Therapak, Claremont, CA, USA). The cards were allowed to dry for 1 h at ambient temperature after which the whole serum spot was punched out with a 6 mm skin biopsy punch (Acuderm, Fort Lauderdale, FL, USA). Each punch was placed in a centrifugal filter with 0.45 μm nylon membrane (VWR, Radnor, PA, USA). One hundred μL of HPLC grade water (JT Baker, Avantor, Allentown, PA, USA) was added to the centrifugal filter containing the punch. The punches were vortexed gently for 10 min then spun down at approximately 10,000 rcf for 2 min. The flow-through was removed and transferred back on to the punch for a second round of extraction. For the second round of extraction, the punches were vortexed gently for 3 min then spun down at approximately 10,000 rcf for 2 min. Twenty μL of the filtrate from each sample was then transferred to a 0.5 mL eppendorf tube for MALDI analysis.

An equal volume of freshly prepared matrix (25 mg of sinapinic acid dissolved in 1 mL of 50% acetonitrile:50% water plus 0.1% TFA) was added to each 20 μL serum extract and the mix vortexed for 30 s. The first three aliquots (2 × 2 μL) of sample:matrix mix were discarded into the tube cap. Three aliquots of 2 μL sample:matrix mix were then spotted onto a polished steel MALDI target plate (Bruker Daltonics, Billerica, MA, USA). The MALDI target was allowed to dry in the biosafety hood before placement in the MALDI mass spectrometer.

Spectral Acquisition

MALDI spectra were obtained using a MALDI-TOF mass spectrometer (Ultraflextreme s/n: 259901.00258 from Bruker Daltonics, Bremen, Germany) equipped with a 2000 Hz SmartBeam laser. Data were acquired in the range from 3 kDa to 30 kDa with positive ion detection in linear mode with the following settings: accelerating voltage set to 25 kV, extraction voltage set to 23.15 kV, lens voltage set to 7 kV, and the delayed extraction time set to 200 ns. The instrument was externally calibrated using the Bruker Protein Standard Mix consisting of insulin, ubiquitin, cytochrome c, and myoglobin.

Eight hundred shot "raster" spectra were collected from 63 pre-defined positions per MALDI spot (63 × 800 × 3 spots per sample) with the fuzzy control for laser power turned off. No evaluation criteria were used to filter out spectra. All filtering and processing of spectra was done post-acquisition.

2. Mass Spectral Processing

Processing of Raster Spectra to Deep MALDI Averages

The spectra were filtered using a ripple filter (simple running averaging of the spectrum) to remove artificial noise resulting from the digital converter. Background, the

variation of spectral intensity on an m/z scale much larger than the peak width, was estimated using asymmetric (25th percentile) running averages with a m/z dependent averaging window width (AWW) in an iterated manner. First, AWW was chosen to be very large to take care of the overall background, and this first estimation of the background generated a first background subtracted spectra. From this, a fine structure background estimate was obtained using a smaller AWW, which again was subtracted again. The background was subtracted for the purpose of finding peaks to be used in alignment. Peaks were detected from the maxima of a convolution of the spectral intensity with a Gaussian function with a half width reflecting the width of the peaks at a given m/z. The threshold for peak detection was set to a signal to noise ratio of 3. The raw spectra (no background subtraction) were then aligned using the calibration points listed in Table S1 using a quadratic function in m/z. Only spectra with a minimum of 20 peaks detected and having used 5 alignment points were considered for inclusion in the average. One hundred forty raster spectra were selected at random to include in the average, resulting in an average spectra of 112,000 laser shots.

Table S1. Points in m/z used to align the raster spectra.

m/z (Da)
4153
6433
6631
8206
8684
9133
11,527
12,572
23,864
13,763
13,882
14,040
15,127
15,869
17,253
18,630
21,066
28,108
28,316

Processing of Deep MALDI Average Spectra

The spectra were background subtracted and normalized using the partial ion current (PIC) windows listed in the table below (Table S2).

Table S2. Normalization windows used in pre-processing the spectra, lower and upper m/z boundaries.

Lower m/z (Da)	Upper m/z (Da)
3231	3301
7106	7178
9996	10,317
15,467	15,771
16,210	16,399

The average spectra were then aligned using the alignment points listed in Table S3 to correct the remaining slight differences in alignment.

Table S3. Alignment points used to align the Deep MALDI average spectra.

m/z (Da)
4154
4361
4711
6432
6631
9420
12,862
13,762
14,039
14,088
14,145
15,128
15,869
17,383
18,272
28,108
28,316

Mass spectral features were defined as regions in m/z by an upper and lower m/z value. Feature definitions were selected by viewing many Deep MALDI average spectra simultaneously and selecting the upper and lower m/z values best encompassing a mass spectral peak across all viewed spectra. Three hundred features were defined for use in classifier development (Table S4).

Table S4. Definitions of mass spectral features.

Lower m/z	Center m/z	Upper m/z
3120.97	3132.28	3143.58
3144.12	3155.69	3167.27
3384.08	3395.16	3406.23
3408.04	3422.03	3436.02
3542.72	3558.63	3574.53
3582.98	3594.26	3605.53
3674.87	3686.35	3697.82
3760.14	3774.13	3788.12
3806.24	3818.82	3831.40
3856.97	3869.35	3881.73
3916.36	3928.94	3941.52
3943.13	3953.50	3963.87
4001.11	4015.00	4028.89
4040.17	4053.15	4066.14
4275.28	4290.71	4306.15
4875.22	4890.97	4906.72
4925.11	4937.26	4949.42
4978.09	4994.69	5011.30
5538.23	5560.98	5583.73
5617.26	5634.92	5652.58
5887.06	5905.52	5923.98
6052.67	6075.36	6098.05
6182.01	6205.21	6228.41

3033.39	3043.08	3052.77
3076.79	3088.26	3099.74
3100.53	3109.47	3118.41
3181.96	3188.28	3194.61
3196.98	3203.71	3210.45
3210.84	3219.94	3229.04
3229.67	3241.25	3252.83
3253.23	3263.29	3273.36
3273.56	3286.24	3298.93
3300.10	3312.52	3324.95
3325.11	3334.68	3344.26
3356.70	3369.99	3383.28
3436.83	3446.59	3456.35
3456.56	3465.51	3474.47
3498.50	3511.87	3525.24
3699.24	3708.03	3716.81
3788.32	3797.18	3806.04
3831.61	3843.99	3856.37
3882.14	3890.89	3899.65
3900.05	3907.70	3915.35
3964.20	3975.77	3987.33
4078.76	4093.64	4108.52
4114.45	4121.20	4127.94
4128.14	4135.99	4143.84
4204.62	4210.25	4215.88
4215.95	4221.58	4227.20
4230.26	4244.74	4259.22
4260.62	4267.38	4274.14
4306.57	4318.72	4330.88
4332.18	4341.37	4350.56
4351.18	4361.06	4370.94
4371.19	4378.76	4386.34
4386.42	4393.22	4400.02
4400.36	4409.29	4418.23
4418.48	4426.49	4434.51
4449.57	4456.23	4462.88
4462.92	4471.86	4480.79
4521.81	4529.99	4538.17
4538.34	4547.75	4557.17
4559.36	4570.55	4581.73
4581.81	4586.52	4591.23
4591.60	4600.18	4608.76
4618.52	4626.79	4635.05
4635.09	4646.56	4658.03
4668.49	4680.92	4693.35
4698.66	4711.22	4723.77
4748.42	4756.67	4764.91
4768.55	4775.84	4783.12
4783.33	4792.26	4801.20
4802.98	4818.65	4834.32
4846.65	4856.27	4865.90

4950.50	4962.87	4975.23
5011.71	5023.51	5035.31
5037.25	5045.01	5052.78
5052.88	5065.50	5078.12
5078.22	5086.70	5095.18
5095.28	5107.75	5120.21
5120.42	5129.61	5138.81
5139.22	5148.31	5157.40
5166.37	5176.18	5185.99
5186.62	5195.87	5205.12
5206.57	5222.14	5237.70
5260.04	5270.05	5280.07
5280.43	5289.47	5298.50
5317.72	5329.70	5341.67
5351.85	5360.73	5369.61
5396.95	5407.02	5417.10
5421.09	5430.67	5440.25
5440.61	5453.46	5466.31
5466.99	5474.16	5481.33
5482.34	5491.64	5500.94
5513.60	5522.04	5530.48
5662.56	5675.33	5688.10
5688.70	5706.56	5724.42
5724.99	5735.14	5745.28
5749.00	5762.00	5775.00
5769.52	5779.10	5788.68
5788.88	5797.16	5805.44
5814.82	5824.40	5833.98
5829.00	5841.50	5854.00
5846.35	5866.50	5886.66
5925.42	5936.84	5948.25
5978.65	5988.93	5999.21
6000.51	6008.02	6015.54
6015.97	6029.24	6042.51
6100.95	6109.32	6117.69
6117.83	6127.05	6136.28
6277.96	6285.65	6293.35
6293.42	6300.21	6306.99
6311.64	6325.69	6339.75
6392.91	6404.05	6415.19
6417.64	6433.26	6448.89
6449.43	6457.72	6466.01
6466.28	6476.88	6487.48
6488.92	6498.60	6508.28
6508.87	6529.73	6550.58
6579.32	6592.59	6605.86
6606.66	6626.41	6646.17
6646.57	6656.45	6666.32
6666.52	6676.70	6686.88
6687.20	6698.07	6708.94
6709.21	6728.64	6748.07

6785.46	6801.92	6818.39
6824.37	6835.85	6847.32
6847.72	6858.90	6870.07
6870.67	6881.05	6891.42
6891.62	6900.70	6909.78
6912.98	6920.66	6928.34
6926.00	6937.00	6948.00
6928.00	6942.50	6957.00
6950.89	6964.26	6977.63
6970.00	6973.50	6977.00
6977.77	6989.03	7000.29
7023.74	7040.65	7057.55
7058.93	7073.87	7088.82
7118.40	7141.12	7163.85
7174.99	7186.67	7198.34
7229.92	7240.81	7251.70
7252.50	7265.78	7279.07
7280.16	7293.73	7307.30
7345.41	7356.19	7366.97
7373.55	7387.02	7400.49
7401.58	7408.92	7416.25
7417.45	7426.93	7436.41
7436.81	7446.59	7456.37
7456.50	7478.98	7501.47
7506.46	7518.23	7530.00
7597.25	7614.12	7630.98
7694.84	7705.91	7716.99
7717.87	7734.18	7750.48
7751.84	7776.03	7800.21
7809.18	7826.04	7842.91
8126.48	8144.14	8161.80
8189.74	8205.00	8220.27
8249.00	8262.67	8276.34
8346.79	8372.73	8398.67
8400.30	8419.35	8438.40
8454.83	8472.27	8489.71
8491.46	8503.54	8515.61
8516.41	8526.59	8536.76
8536.96	8543.75	8550.53
8550.93	8574.58	8598.23
8608.21	8623.67	8639.14
8645.12	8655.40	8665.68
8666.08	8688.23	8710.38
8710.58	8731.23	8751.89
8752.28	8766.55	8780.82
8781.82	8803.67	8825.52
8826.12	8852.66	8879.20
8883.59	8893.87	8904.15
8904.35	8925.80	8947.25
8947.45	8958.92	8970.39
8984.28	8993.64	9003.00

9005.76	9023.07	9040.38
9042.84	9065.99	9089.14
9099.51	9129.35	9159.18
9159.58	9170.35	9181.13
9181.33	9190.11	9198.89
9199.09	9213.36	9227.63
9248.19	9256.96	9265.73
9266.58	9283.28	9299.98
9301.66	9319.42	9337.18
9337.38	9357.24	9377.09
9377.49	9389.47	9401.44
9401.64	9438.56	9475.48
9502.42	9523.27	9544.12
9553.30	9569.27	9585.23
9585.63	9596.71	9607.78
9608.18	9635.42	9662.66
9644.00	9655.00	9666.00
9688.60	9711.45	9734.30
9762.93	9794.85	9826.77
9828.25	9862.61	9896.96
9902.13	9925.28	9948.42
10190.81	10206.39	10221.97
10235.00	10256.74	10278.47
10316.61	10335.66	10354.72
10367.89	10390.04	10412.19
10424.76	10446.32	10467.87
10495.89	10507.13	10518.38
10518.60	10532.01	10545.41
10558.85	10574.44	10590.04
10604.17	10626.82	10649.46
10689.38	10720.71	10752.04
10757.69	10772.29	10786.89
10768.37	10777.35	10786.32
10809.51	10838.94	10868.38
10897.80	10916.69	10935.58
10983.52	11000.18	11016.85
11021.68	11043.26	11064.85
11089.22	11103.28	11117.33
11132.98	11147.56	11162.15
11277.36	11302.94	11328.52
11351.38	11368.14	11384.89
11415.69	11436.82	11457.95
11458.55	11476.97	11495.38
11501.17	11526.51	11551.86
11610.33	11627.89	11645.45
11656.23	11678.78	11701.33
11701.57	11726.48	11751.39
11757.73	11782.33	11806.93
11810.08	11827.25	11844.41
11856.78	11874.44	11892.10
11892.70	11908.27	11923.83

11924.03	11945.78	11967.53
12265.27	12293.61	12321.95
12421.12	12449.86	12478.60
12531.68	12560.61	12589.55
12590.15	12614.39	12638.64
12644.23	12668.08	12691.92
12711.88	12734.33	12756.78
12760.97	12780.53	12800.08
12806.50	12865.74	12924.98
12932.39	12965.13	12997.87
13015.80	13062.37	13108.95
13116.38	13129.95	13139.00
13143.92	13161.28	13178.64
13227.86	13240.86	13253.86
13290.59	13314.34	13338.09
13340.88	13360.63	13380.39
13387.24	13410.82	13434.40
13476.88	13509.03	13541.19
13542.36	13563.60	13584.84
13585.23	13605.30	13625.38
13686.96	13711.90	13736.85
13737.24	13758.09	13778.94
13779.33	13798.43	13817.53
13817.92	13836.43	13854.94
13855.33	13877.75	13900.16
13900.94	13926.08	13951.22
13927.00	13939.50	13952.00
13939.00	13954.00	13969.00
13952.77	13975.96	13999.16
13999.55	14031.90	14064.24
14065.80	14091.92	14118.03
14119.20	14146.87	14174.55
14176.89	14197.93	14218.98
14220.54	14248.21	14275.88
14395.63	14421.89	14448.15
14450.50	14479.92	14509.35
14510.52	14533.90	14557.29
14558.46	14585.94	14613.41
14752.16	14779.33	14806.50
14852.08	14877.14	14902.19
14939.25	14971.99	15004.73
16426.96	16511.73	16596.50
16598.45	16664.52	16730.58
16995.62	17026.80	17057.98
17104.30	17121.88	17139.47
17139.72	17155.18	17170.65
17176.14	17200.08	17224.03
17226.03	17267.43	17308.84
17341.77	17390.41	17439.05
17440.05	17472.35	17504.66
17568.51	17600.69	17632.87

17772.43	17807.18	17841.94
17852.95	17876.12	17899.29
17969.21	18022.01	18074.81
18226.45	18273.65	18320.85
18433.51	18489.23	18544.96
18549.22	18617.73	18686.25
18687.46	18726.44	18765.41
18766.02	18792.21	18818.40
18819.01	18856.46	18893.91
19049.56	19092.01	19134.45
19492.83	19552.08	19611.34
19882.92	19942.61	20002.29
20474.87	20549.17	20623.46
20711.16	20806.16	20901.17
20902.99	20955.06	21007.13
21008.35	21066.81	21125.28
21126.50	21174.91	21223.33
21224.54	21277.22	21329.90
21331.12	21383.19	21435.26
21651.45	21695.30	21739.15
21739.76	21763.51	21787.26
21787.70	21814.48	21841.25

The feature value for each feature was calculated as the integrated intensity of the spectrum across the feature.

Feature values were batch corrected using data from reference samples run on each batch of experimental samples to adjust for possible changes in mass spectrometer performance. Finally, feature values underwent a second normalization using the subset of the 300 defined features listed in Table S5.

Table S5. m/z windows used in the final feature value normalization.

m/z at Center of Feature (Da).
3395
3559
3594
3686
3774
3819
3954
4015
4291
6075
6205
6974
20,549

3. AFP Measurement

Serum AFP levels were measured for each sample using the DAFP00 ELISA kit (R&D Systems, Minneapolis, MN, USA) following manufacturer instructions by ELISA Tech (Aurora, CO, USA). Samples were diluted between 1:2 and 1:5000. The high standard was 20 ng/mL and the standard curve was extended to a low standard of 0.15 ng/mL. A Biotek (Winooski, VT, USA) uQuant ELISA plate reader was used to read at 450 nm with a 630

nm background subtract. Measurements were carried out in duplicate. When the two results varied by more than 35%, the sample was rerun in duplicate until consistent results were obtained for the duplicate pair. The duplicate measurements were then averaged to yield the final result.

4. Generation of Classifications Using an Existing Proteomic Test

It has been observed that a Poor classification from the VeriStrat test occurs infrequently in patients without cancer [23]. Hence, a classification of VeriStrat Poor is likely to indicate the presence of cancer, but with a low sensitivity. To use this information in classification of HCC patients with the spectra generated with the Deep MALDI method, a "VeriStrat-like" classification was obtained by averaging 3 Deep MALDI 800-shot raster spectra in triplicate and applying the VeriStrat classification algorithm to the three averaged spectra. All samples classifying as "VeriStrat-like" Poor were examined to ensure that the classification was not obtained due to the presence of a known confounding peak that can occur with m/z approximately 11.72 kDa. This peak has been observed in patients with severely compromised liver function and hence this check is very important in this patient population. Samples where the "VeriStrat-like" Poor classification was due to a peak at 11.72 kDa were not classified as "VeriStrat-like" Poor.

5. Machine Learning: Development of the Classification Algorithm

Test development was carried out using machine learning with a dropout regularized combination (Diagnostic Cortex®, Biodesix., Boulder, CO, USA) approach [24]. This method was designed to allow reliable estimates of test performance from relatively small development sets in the setting where there are more measured attributes than samples. The method uses an ensemble averaging: the development set was divided into a training set and test set many (625) times. For each training/test split, a master classifier was constructed using only the training set. These 625 master classifiers were ensemble averaged to yield a final classifier. This approach has the advantage that for each training/test split, a portion of the development set is not used in training the classifier. Hence, these "out-of-bag" samples can be reliably classified by the corresponding master classifier. Ensemble averaging results obtained from the training/test splits when a sample is not used in training provides reliable classifications for all samples in the development set, even though they are also all used in classifier training.

First, features of little use in classification were deselected. Subsets of the development set were drawn and k-nearest neighbor (kNN) classifiers created for each individual feature. Each kNN classifier was applied to the training set and a set of spectra from healthy patients. This was repeated for many subsets drawn from the development set. Features which showed little or no ability to identify correctly patients with or without cancer in the training set and to classify healthy patients as cancer-free were discarded. One hundred features were retained for use in classifier training. These are listed in Table S6.

Table S6. Features used in classification (rounded to nearest Dalton for MS features) with measures of their univariate association with presence/absence of HCC in the classifier development set of patients (n = 108, no VeriStrat Poor classification and AFP < 100 ng/mL). Features are sorted according to decreasing AUC.

Feature	*t*-test *p*-value	Mann-Whitney *p*-value	AUC	Tentative Protein ID, if Available*
6901	<0.001	<0.001	0.766	
4792	<0.001	<0.001	0.758	
4267	<0.001	<0.001	0.745	
4053	<0.001	<0.001	0.734	
13,605	<0.001	<0.001	0.732	
5270	<0.001	<0.001	0.730	
4757	<0.001	<0.001	0.725	

13,798	<0.001	<0.001	0.724	
3929	<0.001	<0.001	0.712	
20,549	0.001	<0.001	0.709	Basement membrane-specific heparan sulfate proteoglycan core protein
18,489	0.009	<0.001	0.703	
6881	<0.001	0.001	0.699	
3708	<0.001	0.001	0.697	
AFP	0.001	0.001	0.695	
13,954	<0.001	0.001	0.694	
13,564	0.001	0.001	0.694	
13,758	<0.001	0.001	0.693	Transthyretin
7041	0.001	0.001	0.687	
13,712	<0.001	0.002	0.684	Beta-2-microglobulin (Precursor)
3132	<0.001	0.002	0.683	
9213	0.088	0.002	0.681	
11,477	0.041	0.003	0.676	Immunoglobulin light chain variable region
13,940	0.002	0.003	0.676	Thioredoxin-like protein 5,<br>Cysteinyl glycinated transthyretin
6974	0.018	0.003	0.675	
21,067	0.004	0.003	0.670	Retinol-binding protein
5065	0.002	0.004	0.670	Chromogranin A fragment
20,955	0.008	0.004	0.667	
3109	0.001	0.004	0.666	
14,092	0.003	0.006	0.660	
5474	0.001	0.007	0.657	
14,032	0.004	0.007	0.656	
21,175	0.010	0.007	0.656	
5561	0.007	0.010	0.650	
13,878	0.002	0.011	0.647	Transthyretin, cysteinylated
14,147	0.017	0.012	0.647	
5148	0.170	0.012	0.646	
4530	0.020	0.013	0.644	
10,721	0.012	0.013	0.644	
14,534	0.021	0.018	0.638	
6859	0.023	0.019	0.637	
14,480	0.064	0.020	0.635	Nuclear transport factor 2
14,422	0.038	0.020	0.635	
18,274	0.001	0.022	0.633	
11,874	0.444	0.022	0.633	
10,532	0.042	0.025	0.630	Complement C3
4891	0.012	0.028	0.628	Thymosin beta-10
4600	0.050	0.032	0.625	
10,627	0.593	0.034	0.623	
9170	0.309	0.035	0.623	
5522	0.066	0.036	0.622	
3954	0.016	0.038	0.621	Inter-alpha-trypsin inhibitor heavy chain H4, fragment
5675	0.065	0.040	0.619	
11,726	0.665	0.045	0.617	Beta2-microglobulin
9389	0.045	0.046	0.616	
9066	0.001	0.046	0.616	
4015	0.014	0.049	0.615	
6964	0.822	0.050	0.614	

9863	0.597	0.058	0.610	
6286	0.180	0.060	0.609	
11,437	0.401	0.061	0.609	Serum amyloid A-1 protein
5196	0.073	0.072	0.605	
5867	0.913	0.079	0.602	
9655	0.467	0.081	0.602	
9439	0.079	0.090	0.599	
6937	0.141	0.094	0.598	
6943	0.181	0.104	0.595	
4627	0.196	0.117	0.591	
4647	0.256	0.140	0.586	
13,161	0.335	0.150	0.584	
3286	0.291	0.152	0.583	
4291	0.100	0.196	0.575	
13,509	0.213	0.222	0.571	Serum amyloid A-2 protein
7614	0.508	0.227	0.570	
3891	0.323	0.239	0.569	
19,552	0.273	0.250	0.567	
7409	0.151	0.258	0.566	
8504	0.529	0.260	0.566	
5779	0.259	0.271	0.564	
5906	0.863	0.297	0.561	Fibrinogen alpha chain (577–629)
9190	0.568	0.315	0.559	Haptoglobin a1-chain
13,130	0.012	0.325	0.557	
10,839	0.020	0.337	0.556	Platelet factor 4
7294	0.308	0.409	0.548	
11,946	0.753	0.413	0.548	
17,122	0.309	0.428	0.546	
9257	0.107	0.454	0.544	
9795	0.250	0.561	0.534	
3043	0.551	0.571	0.533	
3335	0.826	0.609	0.530	
8624	0.534	0.613	0.530	
8144	0.782	0.650	0.527	
8731	0.052	0.687	0.524	
5045	0.947	0.739	0.520	
9523	0.586	0.739	0.520	
6008	0.985	0.838	0.512	Elafin
17,876	0.692	0.848	0.511	SUMO-conjugating enzyme UBC9
3797	0.438	0.853	0.511	Submaxillary gland androgen-regulated protein 3B
8853	0.011	0.868	0.510	Secreted Ly-6/uPAR-related protein 1
10,917	0.704	0.888	0.508	
4379	0.661	0.893	0.508	
7826	0.411	0.964	0.503	

*From peer-reviewed literature reviews.

Within each training/test split, the master classifier was created as a strongly regularized combination of atomic, k-nearest neighbor (kNN) classifiers ($k = 9$). Many atomic classifiers were constructed using combinations of one, two, or three mass spectral features and AFP. The ability of these atomic classifiers to identify patients with HCC was tested and only atomic classifiers showing a minimal level of performance on the

classification task were retained; the other atomic classifiers were discarded. The remaining, filtered atomic classifiers were combined using logistic regression regularized by extreme dropout. Subsets of 10 filtered atomic classifiers were selected at random and combined using logistic regression using the training set. This was repeated 100,000 times and the weights for each atomic classifier averaged over the 100,000 dropout iterations. This yielded one master classifier that produces an output between 0 and 1 from the data from each sample.

The ensemble average over the master classifiers from the 625 training/test set splits of the development set (or the ensemble average over master classifiers where a sample is not used in training) generates an output between 0 and 1 for each sample. This can be converted into a binary test by application of a threshold to the continuous output. The classification performance of the tests as a function of the applied threshold can be analyzed using ROC curve methods, such as evaluation of the area under the ROC curve. The threshold value selected to fully determine the final test can be selected according to the clinical requirements of the test. In this case, we wanted to obtain both high sensitivity and high specificity.

Level of liver function can be easily assessed from mass spectral data, with multiple elevated feature values corresponding to lower liver function. This is important, because there may be confounding between level of liver function and presence or absence of HCC. In our available cohorts, this was particularly marked in the UTHSCSA cohort, where samples were collected at time of surgery for liver resection or transplant: the patients with HCC generally had relatively good liver function, while the patients without cancer and undergoing liver transplant generally had poor liver function. To deal with this potential confounding factor, i.e., to avert the danger of developing a test where some or all of the performance in detecting cancer was due to its correlation in the development set with level of liver function, we used an extension of the Diagnostic Cortex filtering component. Instead of requiring only that the atomic classifiers had a minimal level of discriminatory power between cancer and no cancer, we required that they also not classify a set of samples from patients without any liver disease as having cancer. This meant that the master classifiers could not gain a minimal level of performance based on the surrogate of correlation with liver function.

As it has been observed that patients with serum samples classified as Poor by the VeriStrat classification algorithm or with very high AFP (AFP ≥ 100 ng/mL) are very likely to have HCC, patients meeting these criteria ($n = 40$) were assigned a "Cancer" classification. The remaining samples ($n = 108$) in the development set were then used within the machine learning platform for training of a classifier able to identify patients with or without HCC, based on their serum AFP and mass spectral feature values, as explained above.

6. Assessment of Association of Features Used in Test Classification with Presence/Absence of Cancer

The association of each feature used in classification with presence/absence of cancer in the subset of patients used for classifier development (i.e., those with AFP < 100 ng/mL and with VeriStrat classification Good or Indeterminate) was assessed using Student *t*-test, Mann-Whitney test and the area under the curve (AUC) of their receiver-operating characteristic curve.

Figure S1 shows an example of one spectrum obtained from serum collected from a patient with HCC and one spectrum obtained from serum collected from a patient without HCC.

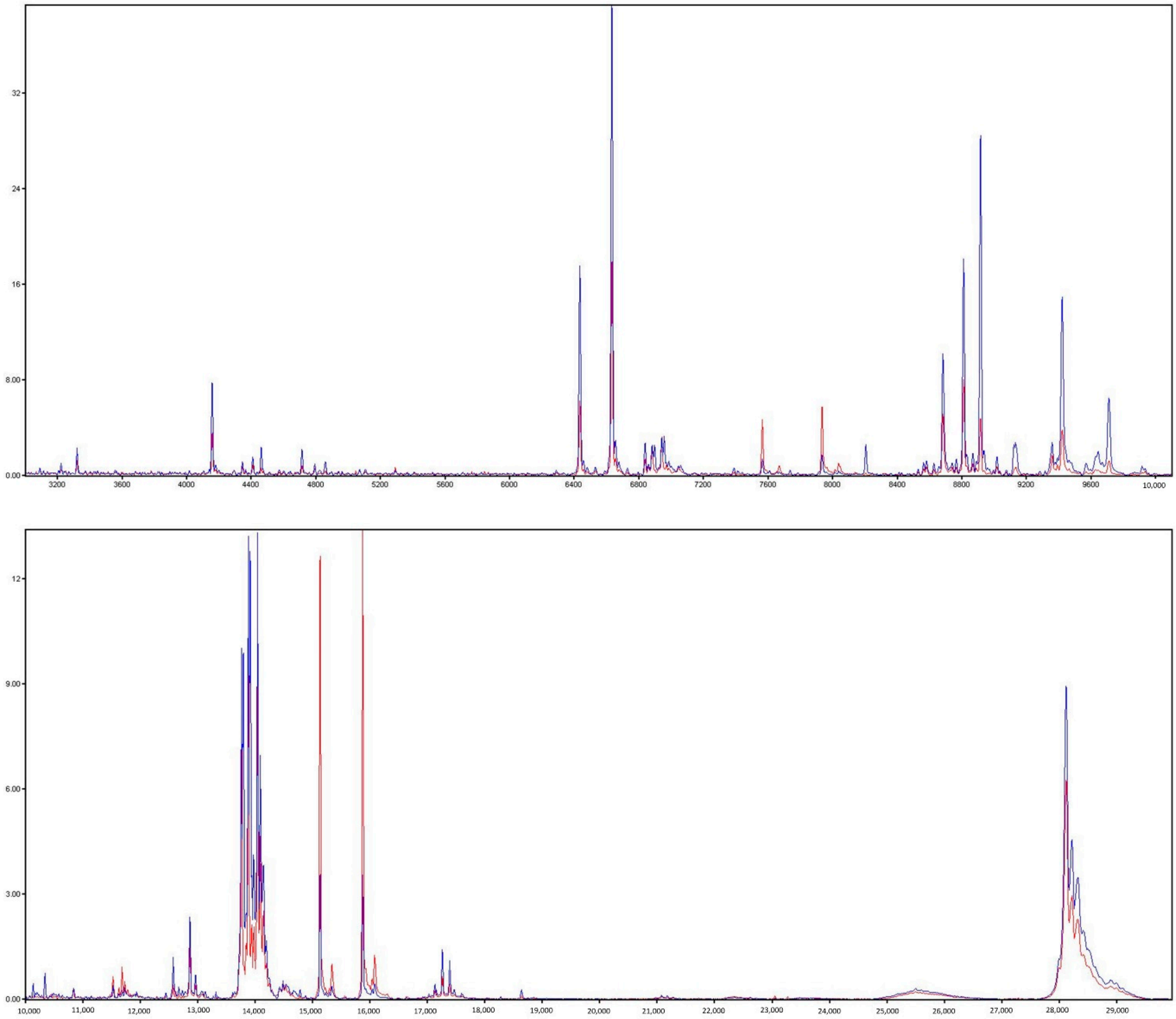

Figure S1. Spectra generated from serum from a patient with HCC (in red) and a patient without HCC (in blue). The first panel shows the spectral range from 3 kDa to 10 kDa and the second panel shows the spectral range from 10 kDa to 30 kDa.

To examine possible importance of pairs of features for classification, we performed a logistic regression for prediction of cancer vs. no cancer using each pair of mass spectral features and their interaction (product of the features). We found 179 pairs of features for which the interaction predicted cancer at a 95% significance level, but where the individual features did not reach this level of predictive performance. For example, for the features 3891 and 6008, the univariate AUCs were 0.57 and 0.51 and neither could predict cancer in univariate logistic regression (p = 0.351 and p = 0.985). When taking the pair of features and their interaction in one logistic regression, again each feature alone had little predictive power (p = 0.774 and p = 0.841), but their interaction showed significant predictive performance (p = 0.0001).

7. Clustering of MS Features and Samples in Figure 2

The heatmap of Figure 2 was produced by hierarchically clustering across features and across samples grouped by presence or absence of HCC. This was performed using the clustergram function within MATLAB R2020a.

8. Additional Results from the Independent Validation Set

Table S7. Accuracy of detection of HCC in Independent Validation (*n* = 97) by grade and stage.

**Grade**	I	12/16 (75%)
	II	16/21 (76%)
	III	11/12 (92%)
	NA	46/48 (96%)
**Stage**	I	9/12 (75%)
	II	12/14 (86%)
	IIIA	18/19 (95%)
	IIIB	2/2 (100%)
	IIIC	10/11 (91%)
	IV	23/25 (92%)
	NA	11/14 (79%)
